# Neuroinflammatory imaging markers in white matter: insights into the cerebral consequences of post-acute sequelae of COVID-19 (PASC)

**DOI:** 10.21203/rs.3.rs-3760289/v1

**Published:** 2024-01-19

**Authors:** Sean Clouston, Chuan Huang, Jia Ying, Zennur Sekendiz, Minos Kritikos, Ashley Fontana, Lev Bangiyev, Benjamin Luft

**Affiliations:** Renaissance School of Medicine at Stony Brook; Renaissance School of Medicine at Stony Brook; Stony Brook University

**Keywords:** Post-Acute Sequelae of COVID-19, Neuroimaging, White Matter, Diffusion Tensor Imaging, Essential Workers

## Abstract

Symptoms of coronavirus disease 2019 (COVID-19) can persist for months or years after infection, a condition called Post-Acute Sequelae of COVID-19 (PASC). Whole-brain white matter and cortical gray matter health were assessed using multi-shell diffusion tensor imaging. Correlational tractography was utilized to dissect the nature and extent of white matter changes. In this study of 42 male essential workers, the most common symptoms of Neurological PASC (n = 24) included fatigue (n = 19) and headache (n = 17). Participants with neurological PASC demonstrated alterations to whole-brain white matter health when compared to controls made up of uninfected, asymptomatic, or mildly infected controls (n = 18). Large differences were evident between PASC and controls in measures of fractional anisotropy (Cohen’s D=−0.54, P = 0.001) and cortical isotropic diffusion (Cohen’s D = 0.50, P = 0.002). Symptoms were associated with white matter fractional anisotropy (fatigue: rho = −0.62, P< 0.001; headache: rho = −0.66, P < 0.001), as well as nine other measures of white and gray matter health. Brain fog was associated with improved cerebral functioning including improved white matter isotropic diffusion and quantitative anisotropy. This study identified changes across measures of white and gray matter connectivity, neuroinflammation, and cerebral atrophy that were interrelated and associated with differences in symptoms of PASC. These results provide insights into the long-term cerebral implications of COVID-19.

## Introduction

In 2019, a severe acute respiratory coronavirus (SARS-CoV-2) caused a pandemic. In some individuals, SARS-CoV-2 causes coronavirus disease (COVID-19), a potentially deadly disease that has caused millions of deaths worldwide predominantly through pathways linked to respiratory, cardiovascular, and neurological damage that the virus causes in vulnerable people ^1, 2^. Changes to central nervous system functioning are a common side effect of several viral infections ^3^, and are a central component of COVID-19 infection ^4^. Correspondingly, COVID-19 infections have been linked with increased depressive symptoms ^5^, cerebrovascular neuropathology ^6^, and increased risk of stroke ^7^. Research among individuals with mild to moderate COVID-19 shows changes in cerebral diffusivity and measures of white matter neuroinflammation ^8, 9^.

There is increasing interest in the lingering symptoms and functional changes that accompany some COVID-19 infections ^10^, especially because these long-term changes can impair activities of daily living ^11^. The Center for Disease Control and Prevention suggest that post-acute sequelae of COVID-19 infection (PASC) develops when acute COVID-19 symptoms last for at least four weeks after infection (https://www.cdc.gov), while the World Health Organization (https://www.who.int) further recognizes that PASC can include emergence of symptoms up to three months following the initial SARS-COV-2 infection that last at least two months without any other explanation ^12^. Pathogenesis of PASC has been linked to SARS-CoV-2 neurotropism even up to 8 months after infection, and with viral-induced coagulopathy, endothelial disruption, systemic inflammation, cytokine overactivation, and neuroglial dysfunction ^13^. The most common neurological complaints in a sample of 10,530 PASC patients at 12 weeks include fatigue (37%), brain fog (32%), memory problems (28%), attention deficit (22%), myalgia (28%), anosmia (12%), dysgeusia (10%), and headaches (15%) ^13^. Symptoms of fatigue, brain fog, executive dysfunction, and slowed response speed in PASC might reflect the presence of axonal ^8, 14-18^ or cortical ^18-22^, an injury that could linger even after COVID-19 has cleared. Follow-up neuroimaging studies of PASC have reported that gray and white matter alterations including changes in white matter mean and axial diffusivity were reported in patients with PASC for as long as 11 months ^23^. While these studies are strongly supportive of the view that PASC might signify the presence of white matter inflammation as measured using diffusion tensor imaging, the prognosis of the disease is unclear.

While ongoing research has used sensitive studies of white matter diffusivity using standard technologies, some symptoms of neuro-PASC could indicate the potential for changes in cortical diffusivity that have not yet been evaluated, while the use of quantitative anisotropy and isotropic diffusion measures have not been examined. The objective of the present study was to study white matter diffusion markers associated with neuroinflammation among individuals with mild to moderate COVID-19 who developed neurological PASC by comparing them to individuals who were never infected with COVID-19 or those who were infected but who never developed PASC.

## Methods

Participants were recruited from a monitoring program for essential workers who work in construction and law enforcement agencies ^24^ with an existing neuroimaging program ^25, 26^. In this program, participants receive an annual personalized monitoring examination that assesses physical, cognitive, and mental health problems. In addition, during the COVID-19 pandemic, the program began to determine COVID-19 status using a barrage of text messaging, phone calls, and letters, and by taking both virtual and in-clinic infection and vaccination histories whenever possible ^27^. In 2020, it became obvious that some individuals were experiencing persistent symptoms for months after initial infections like brain fog and difficulty concentrating so we developed a clinical neuroimaging study to determine whether these neuropsychiatric symptoms being reported alongside PASC were associated with indicators of white matter health. Participants were asked to participate in the neuroimaging program if they fit the eligibility criteria.

For all participants, we collected history of clinical severity (asymptomatic, mild, moderate, and severe), types of acute symptoms during the index COVID-19 case linked with the neuro-PASC diagnosis, the length of time since COVID-19 infection when an infection was present, and the duration of neurological PASC. Participants self-reported their COVID-19 status during their initial phone screening, and we verified information to the best of our ability; we completed a blood draw for COVID-19 antibody testing with all participants. We acquired copies of other positive antibody, PCR, antigen, and molecular test results acquired outside of our clinic, most frequently from local urgent care facilities. We used the date of infections to calculate months between COVID-19 infection and neuroimaging among those who reported a COVID-19 diagnosis.

Neuroimaging analyses included participants who were recruited from three separate groups of participants: neurological PASC: a history of positive COVID-19 testing (antigen, molecular, PCR, or antibody) from 2020–2021 paired with neuropsychiatric symptoms of brain fog and difficulty concentrating lasting ≥ 6 months. Controls included both “uninfected controls” who had a history of negative COVID-19 test results and no COVID-19 symptoms before imaging and “acute COVID-19” controls who reported positive COVID-19 test results but who had an asymptomatic, mild, or moderate presentation of COVID-19 from 2020–2021 but had no evidence of PASC.

We linked COVID-19-related information to demographic and clinical data from our clinical database. All participants were male so demographics included age, education in years, and body mass expressed in kg/m^2^. We measured premorbid crystallized cognition using the Wide Range Achievement Test. As physical functional markers, we included measures of maximal handgrip strength, in 100s of pounds, comfortable walking speed over 12 feet averaged across two trials, and chair rise speed averaged over five trials expressed in rises per second. As measures of fluid cognition, episodic memory was measured using the total score on the Hopkin’s Verbal Language Test, processing speed was measured using the Trails B test and expressed in connections per second, language function was measured using the Boston naming test, working memory was measured using the Symbol Digit Modalities Test, and Verbal Fluency was measured using the Controlled Word Association Test.

### Eligibility Criteria

Due to occupational inclusion, approximately 94% of program participants are male. Since COVID-19 was more severe in men, and because the measures used here are sensitive to participant sex, only males were eligible for this study. Participants were excluded if, during a pre-imaging screening visit, they met any additional exclusionary criteria as discussed below. We were able to verify infection and clinical details for 84.5% of potential COVID-19 cases. Participants whose case status was verified needed to have a body mass index ≤ 40 kg/m^2^ to fit comfortably into the MRI scanner. Participants also needed the capacity to provide informed consent, and a willingness to undergo a blood draw totaling about 100 ml of blood. Participants were fluent in English for neuropsychological testing, and there was no upper age restriction for inclusion, though participants need to be ≥ 18 years of age.

Participants undergoing magnetic resonance imaging (MRI) were excluded if they presented with a history of psychosis, a history of stroke, a history of serious head trauma as defined by loss of consciousness accompanied by confusion, slurred speech, or amnesia, or other neurological disorders such as epilepsy. We also excluded participants with a history of brain cancer, chronic autoimmune diseases, or heart failure, and participants currently in renal failure or receiving dialysis, those who had a myocardial infarction in the past year, and/or an indication of unmanaged diabetes, and those who had evidence of severe liver disease or hepatitis. Participants receiving cognitively active medications, or anti-inflammatory, or immunomodulatory drugs were also excluded. Participants with claustrophobia, embedded ferromagnetic metal implants, pacemakers, shrapnel, wires, and/or other MRI-unsafe surgical implants were excluded.

### MRI Acquisition

All images were acquired on a 3T Siemens Biograph mMR scanner (V.VE11P) using the vendor-provided 20-channel head/neck coil. Diffusion MRI images were acquired using a state-of-the-art multi-band diffusion-weighted imaging sequence ^28, 29^ (obtained via C2P Center for Magnetic Resonance Research, University of Minnesota) with TE/TR = 121.4/6300 ms, voxel size = 2x2x2 mm^3^, multi-band factor = 3. A multi-shell diffusion scheme was used with multiple b-values = 1000, 2000, 3000, and 4000 s/mm^2^ with diffusion sampling directions 64, 32, 32, and 32, respectively. T1-weighted magnetization-prepared rapid gradient echo (MPRAGE) images were also acquired (TE/TR/TI = 2.49/1900/900 ms, flip angle = 9°, isotropic voxel size = 0.9x0.9x0.9 mm^3^, grappa factor = 2, Turbo factor = 192).

### MRI image processing

Brain parcellation was performed with FreeSurfer (7.3.0) using T1-MPRAGE images. Two subjects were removed from the analysis because, upon visual inspection, there was evidence of chronic cerebral infarctions. The diffusion MRI images were processed using DSI Studio (V.06142023). FSL’s eddy was used to correct for eddy current distortion. We calculated fractional anisotropy (FA), mean diffusivity (MD), axial diffusivity (AD), and radial diffusivity (RD) using diffusion tensor imaging with diffusion images acquired with b-value = 0; 1,000 s/mm^2^. To generate the spin distribution function ^30^, Q-space diffeomorphic reconstruction ^31^ was performed in the MNI (Montreal Neurological Institute) space with a diffusion sampling length ratio of 1.25. Output resolution in diffeomorphic reconstruction was an isotropic 2 mm. The model-free technique separates isotropic from anisotropic diffusion, thereby reducing partial volume effects and crossing fibers. This approach has been shown to have improved reliability against edema^32^ and crossing fibers^33^. It allows the quantification of isotropic diffusion (ISO, isotropic value of the spin distribution function). Quantitative anisotropy (QA) was extracted as a local connectome fingerprint ^34^, and used in connectometry analyses. Whole-brain average white matter diffusion parameters were calculated using masks generated by combining left and right cerebral white matter in the FreeSurfer DKT atlas. Cortical thickness and cerebral volume were extracted from the T1MPRAGE images using the standard pipeline in Freesurfer. All image analyses were performed on a Mac mini with Apple Silicon M2 Pro (32GB memory) running Ventura 13.5.

### Statistical Analyses

For gross comparisons between group characteristics, the Fisher exact test was utilized for categorical variables, while Welch’s t-test was utilized for continuous variables. To report effect sizes after adjustment for covariables we used generalized linear modeling and estimated standardized beta coefficients (b). All modeling assumptions were tested, and models were adjusted for potential unmatched confounders. When examining symptoms-based correlations within COVID-19-infected individuals, models adjusted for age, and intracranial volume. When examining cognitive outcomes, we additionally adjusted for schooling and estimated premorbid cognitive ability. Heat maps were generated, and estimated effect sizes were overlaid to aid in interpretation.

Correlational tractography was used to examine white matter tractography in COVID-19-infected versus uninfected participants and to compare those with acute COVID-19 to those with PASC. We used a nonparametric Spearman partial correlation to derive the correlation because of the small sample size and the potential for non-Gaussian distributions. As an effort to show differences in effect size, we reported images with a length threshold of 15 voxels (where T = 1), that were tracked using a deterministic fiber tracking algorithm with whole brain seeding. Generated tracts were then filtered by topology-informed pruning with 16 iterations. We use the area under the receiver-operating curve (AUC) to indicate the extent to which neuroimaging measures differed between diagnostic categories.

Since cerebral atrophy can indicate increased severity and poorer prognoses in neurological conditions, we examined correlations between gray and white matter parameters to determine if any white matter changes were related to neurodegenerative differences. Volumetric estimates were adjusted for total intracranial volume in all models.

Statistical significance was determined using a two-tailed p-value (a = 0.05) and adjusted for the false discovery rate (FDR) ^35^. FDR in tractography analyses was estimated using a total of 4,000 randomized permutations applied to the case label to obtain the null distribution of the tract length; tracts significant after adjusting for multiple comparisons (FDR = 0.05) were reported. Group-wise analyses were performed using Stata MP/17 (StataCorp), and Tractography was analyzed using DSI-Studio (dsi-studio.labsolver.org).

### Sensitivity Analyses

Some factors might influence vulnerability to other cerebrovascular or neurodegenerative diseases, so we examined the potential for diffusion and volumetric parameters to relate to vaccination status or APOE4 status.

### Ethics

The [Institution] internal review board reviewed all study procedures. Participants provided informed written consent to participate in this study.

## Results

### Participant Characteristics

**Figure 1:**
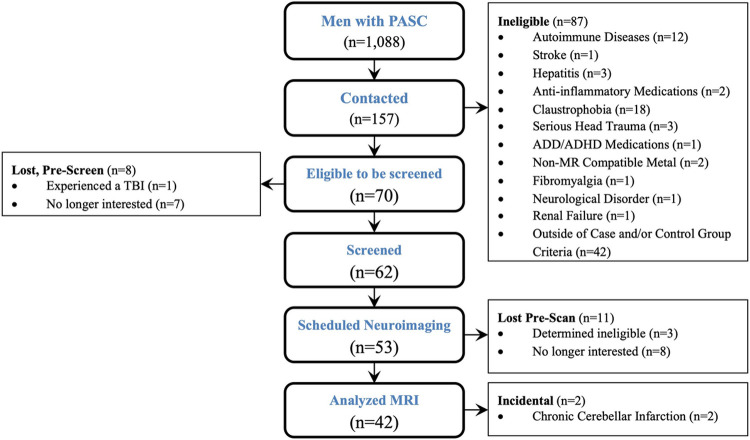
Post-Acute Coronavirus Disease, 2019, Neuroimaging Consort Table

Only 70 of 788 PASC patients met the study's eligibility requirements, and 42 participants (24 participants with PASC and 18 controls), were included in this study (Figure 1). As shown in Table 1, the average age, education, and cognitive ability metrics were matched between PASC participants and controls, but PASC participants had more severe clinical disease and more variable symptoms during their acute infections. Differences between infected and uninfected controls are shown in Supplemental Table 1. The average COVID-19 antibody levels were similar between PASC and COVID-19 acutely infected controls. Not shown in Table 1, individuals with PASC developed a range of common symptoms, with more than half of all PASC participants reporting persistent fatigue (n=19), either cough or shortness of breath (n=18), headache (n=17), joint or muscle pain (n=16), chest pain (n=14), fever (n=13), or either brain fog or loss of taste or smell (n=12). The least common persistent symptoms in this study were nausea (n=1) and weight loss (n=4).

### Whole-brain analysis

In Table 2, we tabulated and identified whole-brain white matter averages of MD, RD, and ISO were found to be significantly increased in participants with PASC, and that average FA was significantly lower in participants with PASC. Whole brain gray and white matter FA and ISO, cortical volume and thickness, and white matter RD and MD values survived FDR correction.

Between-group comparisons of FA, MD, AD, ISO, QA, and RD diffusion parameters the whole white matter was stratified by PASC status (Supplemental Figure 1). These results suggested that diffusion parameters differed between groups, even at the whole-brain level, and that accuracy to detect PASC was highest for FA with an AUC of 0.80 and accuracy of 71.4%. Inter-measure correlations are shown in Supplemental Table 1.

### Correlational Tractography

**Figure 2:**
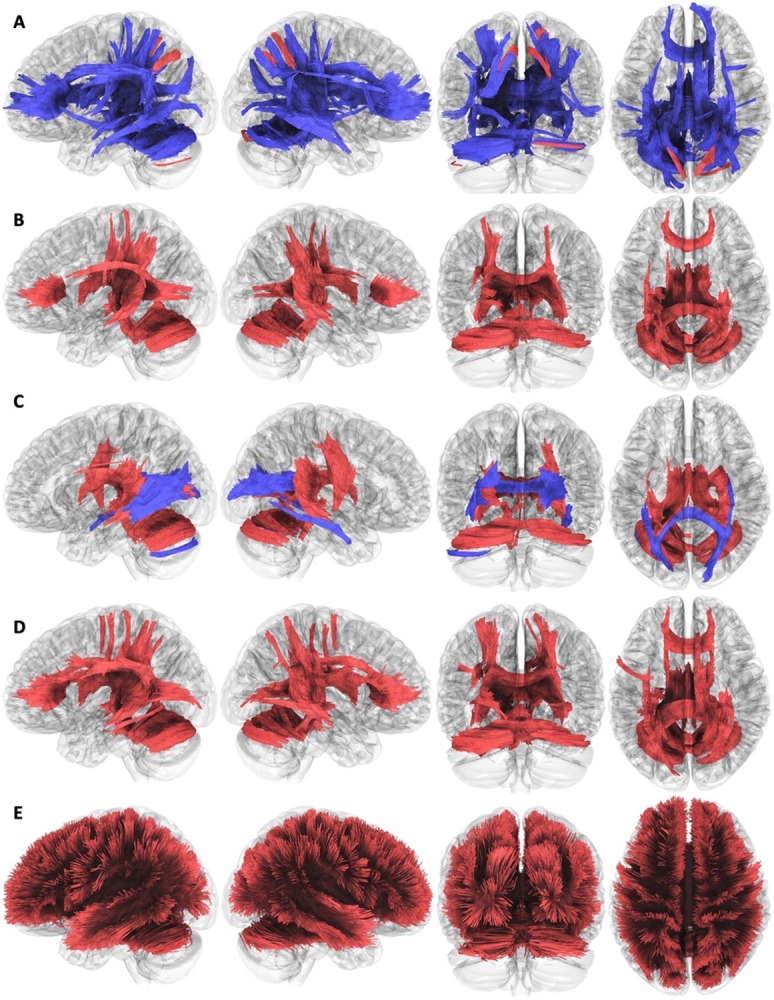
Correlational tractography comparing participants with post-acute sequelae of coronavirus disease with controls. Tracts are shown when they are statistically significant as defined by having an estimated false discovery rate < 0.05 (T≥1). Blue tracts are shown to indicate the presence of decreases in the outcome of interest, while red shows tracts where the outcome has increased. Each panel shows four different directional views of the brain, and panels differ by neuroimaging outcome: A) Fractional Anisotropy; B) Mean Diffusivity; C) Axial Diffusivity; D) Radial Diffusivity; E) Isotropic Diffusion.

To examine the scope of white matter changes, we performed correlational tractography to identify white matter tracts with a diffusion parameter correlated with the group variable (i.e., PASC versus controls). Figure 2 shows that diffusion measures displayed global changes in white matter tractography globally (increases shown in red, decreases in blue) that survived FDR adjustment. Whereas most diffusion measures (Figure 2, Panels A-D) identified regional changes in the cingulate, cerebellum, and parietal lobes as well as in the subcortical regions, ISO was found to increase throughout the entire white matter (Figure 2, Panel E). Whole-brain cortical gray matter averages of MD, RD, and AD were also studied but none had tractography results that were found to be significantly different between the PASC and control groups.

### COVID-19 symptoms

Next, we examined Spearman’s correlation coefficients to test the hypothesis that specific acute COVID-19 symptoms in cases would be associated with neuroimaging measures (Table 3). After adjusting for intracranial volume and age, we found that neurological symptoms including nausea, headache, and fatigue were associated with differences in white matter connectivity. The presence of brain fog was associated with evidence of reduced ISO whereas nausea, joint pain, and fatigue were associated with increased ISO. Lower cortical thickness was associated with headache, chest pain, wheezing, shortness of breath, and nausea.

### Regional Fractional Anisotropy Signature

**Figure 3:**
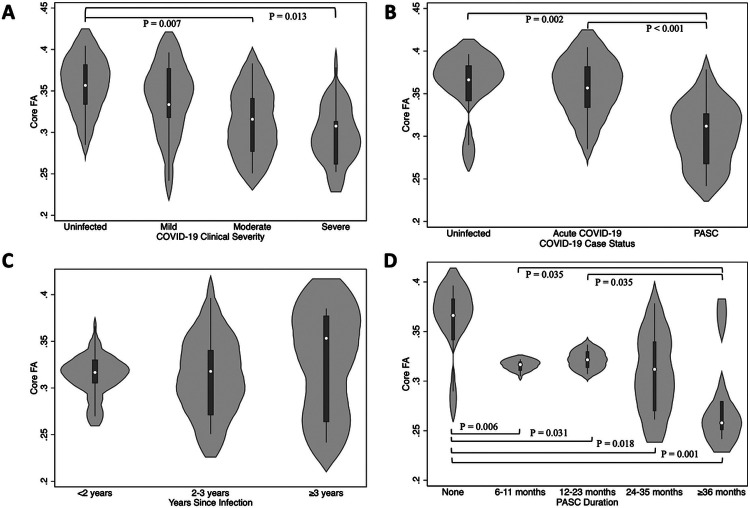
Violin plots showing Core Fractional Anisotropy levels stratified by Acute Coronavirus disease, 2019, and post-acute sequelae of Coronavirus disease, 2019, characteristics. Nominal p-values, derived from multiple t-tests, are reported. Core FA: Core Fractional Anisotropy. Panels showing A) Acute COVID-19 severity, B) Case status (no COVID, acute COVID only, and post-acute sequelae of COVID-19), C) Grouped years since initial infection, D) duration of post-acute sequelae of COVID-19.

**Figure 4:**
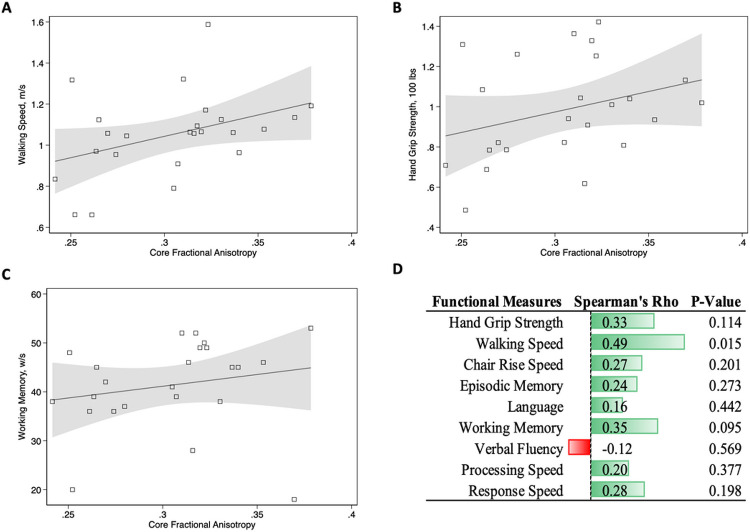
Scatter plots and trend lines showing associations between physical and cognitive functioning in participants with post-acute sequelae of Coronavirus disease, 2019.

To further examine which regions of the brain were most affected we performed correlational tractography results using different thresholds (Supplemental Figure 2 and 3). These results show that the tracts consistently found to have the strongest effect included the left and right fornix, Forceps Minor, and Tapetum. We further estimated tract FA averages derived from the tractographic analyses shown in Figure 2, Panel A, and termed that measure “CoreFA”. Core FA had the highest effect size than any of the whole-brain measures shown in Table 2 (b = −0.59, P<0.001, AUC=0.81). Next, we examined the hypothesis that clinical indicators of COVID-19 severity were associated with changes to Core FA (Figure 3). Results showed that Core FA was increased in individuals with more clinical severity of acute COVID-19 (Panel A), the presence of PASC (Panel B), lengthier periods between infections and ongoing symptoms (Panel C), and lengthier duration of infections (Panel D). Variability around Core FA was highest when participants were further away from their diagnosis, while those whose neuro-PASC symptoms persisted for ≥3 years showed the strongest changes in Core FA. Finally, we examined the association between Core FA and functional symptoms in PASC (Figure 4, with overall results in Panel D). Results suggested that Core FA was associated with slower walking speed (Panel A), while results with possible trends are shown in Figure 4, Panels B and D.

Since prognosis is a question of some pressing importance, we examined the degree of association between white matter alterations and gray matter structural outcomes in COVID-19-infected individuals (Table 4). These results identified moderate to strong associations between reduced white matter FA and reduced cortical and subcortical volume and cortical thickness. Increases in white matter ISO, QA, and gray matter ISO were also associated with reduced cortical thickness. Additionally, white and gray matter MD, RD, and AD were associated with cortical volume, subcortical gray matter volume, whole-brain gray matter volume, and whole-brain volume among those who reported COVID-19.

### Sensitivity Analyses

Whole-brain diffusion parameters and white/gray matter volumes were not found to have a noticeable association with vaccination status, or APOE4 status aside from associations with COVID-19 severity (data not shown). Comparisons of the two control subgroups used multiple regression analysis to adjust for the effects of age and school years in all study participants, and the group residuals were used for t-tests, which did not identify any difference in diffusion parameters (data not shown).

## Discussion

The presence of neurological PASC is a new problem emerging after acute COVID-19 and may persist for years after initial infections. Replicating and expanding on prior neuroimaging work among COVID-19 patients, the present study identified pronounced changes in white matter diffusion parameters in PASC patients compared to both COVID-19 naive and COVID-19 infected participants without PASC. Using correlational tractography, we elucidated the presence of widespread neuroinflammation, with findings indicative of symmetric axonal injury. Elevated values of white matter MD, RD, and ISO coupled with diminished FA values and insignificant AD and QA changes observed in PASC patients are strongly suggestive of axonal injuries with neuroinflammation ^36-38^. Indeed, the robustness of the FA parameter, particularly the whole-brain white matter average FA value, in differentiating PASC from controls (AUC > 0.8) underscores its potential clinical significance. For example, when considering the severity of the acute COVID-19 infection, individual measures were able to differentiate COVID-19 controls including those who were never infected and those who were only acutely infected from those with neurological PASC. The robustness of the AUC values and logistic regression prediction accuracy highlights their potential diagnostic utility, hinting at a promising avenue for more precise and early detection of this condition. Our results bridge the current knowledge gap by highlighting the pervasive inflammatory landscape for individuals with neurological PASC.

Our work replicates and expands on analyses showing that COVID-19 can induce changes in white matter health. Prior work has reported reduced axonal density in hospitalized COVID-19 patients up to one year after recovery ^39^, while others have noted changes consistent with neuroinflammation in hospitalized COVID-19 patients ^16^. In the largest study to date, researchers found that individuals with mild to moderate COVID-19 appear to show changes in cerebral diffusivity and measures of neuroinflammation ^8^. The present work expands upon this earlier research and suggests that the presence of white matter dysfunction likely indicates the potential for PASC in patients infected with COVID-19, and further notes that the presence of FA dysfunction, in particular, is associated with a wide array of neurological PASC symptoms including fever, headache, fatigue, and pain, while we also found that white matter QA and white and gray matter ISO were strongly associated with brain fog symptoms.

We also found that decreases in whole-brain white matter FA was associated with reduced cortical thickness. Similar results showed that increases in white matter ISO, QA, and gray matter ISO were also associated with cortical thickness. Similarly, we found that several white matter and gray matter measures were also associated with cortical, subcortical, and whole-brain gray matter volume, as well as with reduced whole-brain volume and increased cerebrospinal volume. Cortical thickness and cerebral brain volume are established biomarkers for neurodegenerative diseases including Alzheimer’s and related dementias ^40^. Future work should examine the location of structural damage to help determine the potential for chronic functional limitations.

Controls with a history of acute COVID-19 and individuals lacking COVID-19 exposure did not show marked differences in the diffusion parameters evaluated here. This observation strengthens the premise that the neurocognitive sequelae in PASC, often recognized by the presence of brain fog, are intricately tied to neuroinflammation. The absence of notable differences between never-infected controls and those recovered from COVID-19 further accentuates the pivotal role of neuroinflammation in PASC-related cognitive deficits.

Studies have noted that some symptoms of PASC, like anosmia, anxiety, and brain fog, eventually dissipate in a majority of PASC patients in the months or years after initial infections though others, like fatigue, might show a pattern of relapse potentially with additional ^41^. Indeed, the brain's capacity for neuroplasticity raises the question of whether observed changes are permanent, or instead reflect a potential for recovery or adaptation over time. The potential that the resolution of symptoms reflects a reversal of diffusion parameters upon symptom resolution could provide some indication that the brain is resilient, in the long term, against these effects. Longitudinal studies will be paramount to tracking the progression or potential resolution of these neural changes over time, setting the stage for more comprehensive therapeutic interventions and a deeper understanding of PASC's long-term implications.

Findings inherent here might help to isolate the mechanisms of neuro-PASC. Changes in FA are often thought to measure the degree of axonal injury and decreased FA often indicates the presence of neuroinflammation. Increased AD often indicates an increase in axonal density consistent with an increased axonal inflammation. Increased RD in white matter suggests that neuroinflammation might be contributing to axonal demyelination. Changes in cortical MD have been associated with cerebral astrocytosis in familial Alzheimer’s disease ^42^. Gray matter evaluation presented a distinct landscape. Significant differences in ISO and cortical gray volume in PASC echo the potentially lasting severe findings in prior PASC studies ^22^, and might reflect the significant correlations found between ISO and FA with cortical and whole-brain volumetry reported here. Cortical and subcortical atrophy are central indicators of neurodegenerative disease that often result in cognitive, behavioral, and physical functional changes and can reduce independence. The observed alterations in diffusion parameters, especially within the white matter, pave the way for a deeper understanding of the possible cellular or molecular mechanisms responsible for chronic PASC. However, collectively, these observations imply that inflammatory changes in neuro-PASC could lead, at least for some individuals with neuro-PASC, to discernible cerebral atrophy. The presence of demyelination and neurodegeneration likely belies the potential for neuro-PASC symptoms to persist and raise the potential for symptoms to worsen with time.

### Limitations

This study has several important limitations. First, one of the most salient limitations of this study is the relatively small sample size, which reduces the statistical power to detect anything but the largest associations. While the focus on outcomes among males is important because COVID-19 is more severe in males ^43^, a lack of females in our cohort limits the generalizability of these findings. Second, the cross-sectional nature of this study limits our ability to conclude causality, or the temporal progression of changes observed in PASC patients. Longitudinal studies are needed to monitor the trajectory of these brain alterations over time both among individuals with mild to moderate acute COVID-19, and among individuals who develop PASC could help to elucidate the persistence of changes. Third, our study alludes to neuroinflammation as a potential cause for the observed brain changes. However, we lack any direct measures identifying specific systemic or CNS-specific inflammatory markers in the brain, making this labeling speculative. Follow-up work including work using molecular neuroimaging is now emerging and may help to determine the specific types of neuroinflammation identified here ^44^.

### Implications

Currently, neurological PASC is a syndrome that includes several symptoms including fatigue or brain fog. In the past, conditions with these symptoms have been difficult to identify or to understand using both biomarkers, structural neuroimaging, and cognitive or physical functional changes. Our study similarly showed that cognitive and physical functional symptoms were mild or lacking for a wide range of test outcomes, despite evidence of a substantial neuroinflammatory signature. The results of this study imply that we might be able to positively diagnose neurological PASC using a neuroimaging-based signature in the white matter with a high degree of accuracy. Indeed, the presence and importance of FA and ISO imply the potential for widespread neuroinflammation as a central mediator of these symptoms. Given the changes evident here, therapeutic strategies targeting neuroinflammation might prove beneficial for PASC patients. Interventions that counteract neuroinflammatory processes could be a significant step forward in managing and alleviating the syndrome's debilitating symptoms.

## Figures and Tables

**Table 1:** Sample characteristics for all neuroimaged participants, stratified into post-acute sequelae of COVID-19 and control groups.

Sample Characteristics	Whole Sample	Post-Acute Sequelae of COVID-19	COVID-Infected and Uninfected Controls	D, P
	N=42	n=24	N=18	
Age	59.8 (7.9)	61 (8.5)	58.9 (6.8)	0.27, 0.392
Education, Years	16.2 (2.1)	16.1 (1.8)	16.4 (2.7)	−0.14, 0.683
Premorbid Cognition	99.6 (11.3)	99.3 (9.8)	101.2 (13.1)	−0.16, 0.625
Body Mass, kg/m2	29.4 (3.9)	29.3 (4.4)	30.1 (3)	−0.21, 0.496
COVID-19 Indicators				
Months Since Infection*	30.1 (9.4)	28.8 (9.9)	33.5 (7.5)	−0.53, 0.213
COVID-19 Severity	1.5 (1)	2 (0.7)	0.8 (0.9)	1.5, 0
PASC Symptom Burden	5 (4.1)	7.3 (3.5)	1.9 (2.9)	1.69, 0
COVID-19 Antibody Levels	3.1 (3.1)	3.4 (3.3)	2.5 (2.8)	0.31, 0.33
Physical Functioning				
Grip Strength	10.1 (2.2)	98.2 (25.3)	107.4 (15.7)	−0.43, 0.167
Walking Speed	1 (0.2)	1.1 (0.2)	1 (0.1)	0.25, 0.429
Chair Rise Speed	5.1 (1.4)	49.6 (13.7)	52.6 (15.3)	−0.21, 0.523
Cognitive Functioning				
Episodic Memory	21.7 (4.8)	21.4 (0.9)	21.7 (21.4)	−0.06, 0.855
Processing Speed	1.4 (0.5)	1.3 (0.4)	1.6 (0.5)	−0.57, 0.093
Language	29.5 (1)	29.4 (1.2)	29.7 (0.6)	−0.28, 0.366
Working Memory	43 (9)	41.3 (9.3)	44.3 (8.2)	−0.34, 0.294
Verbal Fluency	41.3 (11.2)	39.8 (9.5)	44.2 (12)	−0.41, 0.225

**Note:** COVID-19: coronavirus disease 2019; Std.: standardized; Diff.: difference; P: p-value.

**Table 2:** Means and standard deviations for diffusition parameter stratified by post-acute sequelae of novel coronavirus 2019 as compared to all controls, rank-ordered by multivariable-adjusted standardized regression coefficients

Neuroimaging Indicators	Post-Acute Sequelae of COVID- 19 Mean (SD)	COVID-Infected and Uninfected Controls Mean (SD)	Std. Coef., P	AUC, Sensitivity, Specificity
White Matter Fractional Anisotropy^a^	0.31 (0.01)	0.32 (0.01)	***−0.54, 0.001***	0.80, 0.75, 0.67
Gray Matter Isotropic Diffusion^a^	0.33 (0.04)	0.29 (0.04)	***0.50, 0.002***	0.72, 0.63, 0.72
Gray Matter Volume^b^	0.63 (0.04)	0.66 (0.05)	***−0.47, 0.003***	0.64, 0.71, 0.61
White Matter Isotropic Diffusion^a^	0.38 (0.05)	0.35 (0.04)	***0.46, 0.004***	0.71, 0.54, 0.83
Cortical Volume^b^	0.46 (0.03)	0.48 (0.04)	***−0.45, 0.004***	0.63, 0.71, 0.67
White Matter Radial Diffusivity^c^	0.73 (0.05)	0.69 (0.04)	***0.45, 0.004***	0.75, 0.67, 0.78
White Matter Mean Diffusivity^c^	0.86 (0.05)	0.83 (0.04)	***0.41, 0.008***	0.73, 0.63, 0.78
Cortical Thickness^d^	2.41 (0.07)	2.45 (0.07)	***−0.34, 0.021***	0.67, 0.67, 0.67
Cerebrospinal Fluid Volume^b^	0.13 (0.03)	0.11 (0.03)	**0.31, 0.031**	0.66, 0.63, 0.78
White Matter Axial Diffusivity^c^	1.12 (0.05)	1.10 (0.04)	0.26, 0.054	0.66, 0.54, 0.72
Whole Brain Volume^b^	0.73 (0.03)	0.74 (0.03)	−0.22, 0.088	0.62, 0.67, 0.50
White Matter Quantitative Anisotropy^a^	0.15 (0.02)	0.15 (0.02)	0.19, 0.120	0.61, 0.46, 0.83
Gray Matter Radial Diffusivity^c^	1.11 (0.09)	1.07 (0.11)	0.14, 0.202	0.63, 0.63, 0.61
Gray Matter Mean Diffusivity^c^	1.17 (0.09)	1.14 (0.11)	0.13, 0.217	0.62, 0.63, 0.61
Subcortical Volume^b^	0.06 (0.01)	0.06 (0.01)	−0.11, 0.244	0.57, 0.63, 0.61
Gray Matter Axial Diffusivity^c^	1.30 (0.09)	1.26 (0.11)	0.11, 0.253	0.61, 0.63, 0.61

a unitless

b liter

c* 10^−3^

d millimeters

**Note:** Std.: standardized; Coef.: Coefficient; AUC: Area under the receiver operating curve.. Standardized regression coefficient comparing PASC to control groups as estimated using generalized linear modeling to account for intracranial volume and age. Nominal p-values are reported; **bold** typeface shows which tests are nominally significant; ***bold italicized*** typeface shows which tests remained statistically significant upon adjusting for the false discovery rate.

**Table 3:** Heatmap correlation table showing standardized regression coefficients linking COVID-19 symptoms with neuroimaging parameters

		Fever	Fatigue	Headache	Cough	Chills	Joint or Muscle Pain	Chest Pain	Wheezing	Sore Throat	Anosmia	Gastro- Intestinal	Shortness of Breath	Nausea	Dizziness	Anxiety	Brain Fog	Congestion	Weight Loss
White Matter	Fractional Anisotropy	***−0.41***	***−0.62***	***−0.66***	***−0.47***	0.02	***−0.47***	***−0.47***	***−0.42***	−0.25	−0.35	−0.26	−0.37	***−0.40***	−0.33	−0.23	−0.12	−0.31	−0.08
	Isotropic Diffusion	0.14	***0.43***	0.29	0.20	−0.14	***0.43***	0.17	0.22	***0.39***	0.19	0.28	0.22	***0.71***	0.12	−0.31	***−0.90***	−0.07	−0.34
	Radial Diffusivity	***0.47***	***0.52***	***0.72***	***0.55***	0.07	***0.49***	***0.50***	***0.42***	0.30	0.33	0.29	***0.44***	***0.49***	0.21	0.02	0.13	0.22	−0.06
	Mean Diffusivity	***0.43***	***0.45***	***0.66***	***0.49***	0.09	***0.46***	***0.44***	***0.38***	0.28	0.28	0.26	***0.40***	***0.55***	0.15	−0.04	0.10	0.16	−0.10
	Axonal Diffusivity	0.30	0.27	***0.46***	0.33	0.11	0.35	0.28	0.25	0.19	0.16	0.18	0.28	***0.66***	0.03	−0.16	0.03	0.02	−0.15
	Quantitative Anisotropy	−0.07	0.19	0.10	0.01	−0.07	0.28	0.00	0.06	0.22	0.10	0.12	0.08	0.27	−0.20	−0.30	***−1.01***	−0.13	−0.28
Gray	Isotropic Diffusion	0.16	***0.42***	0.26	0.20	−0.15	***0.40***	0.15	0.17	***0.39***	0.17	0.26	0.24	***0.91***	−0.12	−0.29	***−0.82***	−0.08	−0.34
	Radial Diffusivity	0.25	0.20	0.22	0.35	−0.05	0.18	0.25	0.18	0.20	0.17	0.11	0.23	−0.26	−0.33	−0.14	0.18	0.20	−0.16
	Mean Diffusivity	0.25	0.19	0.22	0.35	−0.04	0.18	0.24	0.18	0.20	0.16	0.10	0.23	−0.23	−0.32	−0.15	0.18	0.19	−0.16
	Axonal Diffusivity	0.25	0.19	0.21	0.34	−0.01	0.18	0.22	0.17	0.19	0.13	0.08	0.22	−0.19	−0.29	−0.17	0.19	0.17	−0.15
Volumetrics	Gray Matter	−0.25	***−0.37***	***−0.56***	−0.31	−0.02	−0.17	−0.26	−0.33	−0.14	***−0.45***	−0.03	−0.25	0.20	−0.16	−0.22	−0.24	−0.14	−0.18
	Cortical Volume	−0.10	−0.26	***−0.46***	−0.34	0.03	−0.07	−0.17	−0.22	−0.01	−0.30	0.05	−0.15	0.11	−0.13	−0.11	***−0.41***	−0.14	−0.19
	Cortical Thickness	−0.10	−0.28	***−0.70***	−0.34	−0.10	−0.14	***−0.40***	***−0.44***	−0.07	−0.21	−0.22	***−0.42***	***−0.44***	−0.15	0.31	0.04	0.07	0.28
	Cerebrospinal Fluid	0.07	0.10	0.30	0.29	***0.40***	−0.05	0.28	0.24	0.17	0.12	0.12	0.34	0.00	0.17	0.06	0.34	0.05	0.15
	Sub-Cortical	***−0.42***	−0.13	***−0.45***	−0.23	−0.26	0.00	***−0.49***	−0.30	−0.08	***−0.38***	−0.16	−0.28	***0.70***	0.03	0.02	−0.13	0.04	0.00
	Whole Brain	−0.31	−0.23	−0.23	−0.26	−0.05	−0.10	−0.32	−0.26	−0.18	***−0.38***	−0.05	−0.13	0.37	−0.11	−0.30	−0.26	−0.25	−0.24

Note: Standardized regression coefficients reported comparing PASC to control groups as estimated using generalized linear modeling to account for intracranial volume and age. Nominal p-values are reported in black typeface when the p-value exceeds 0.10; bold typeface shows which tests are nominally significant (two-tailed a=0.05); *bold italicized* typeface shows which tests remained statistically significant upon adjusting for the false discovery rate.

**Table 4.** Heatmap showing standardized regression coefficients from generalized linear models examining correlates of white and gray matter health linked with volumetric measures in COVID-19-infected participants with and without Post-Acute Sequelae of COVID-19

		White Matter					Gray Matter				
		ISO	QA	MD	RD	AD	FA	MD	AD	RD	ISO
Volumetrics	CTX	***−0.44***	***−0.33***	−0.24	−0.26	−0.17	***0.32***	−0.03	−0.02	−0.03	***−0.37***
	CTV	−0.19	0.01	***−0.49***	***−0.50***	***−0.37***	***0.58***	***−0.48***	***−0.47***	***−0.48***	−0.19
	SCV	−0.11	0.07	***−0.48***	***−0.55***	−0.25	***0.39***	***−0.66***	***−0.64***	***−0.66***	−0.12
	GMV	***−0.33***	−0.05	***−0.44***	***−0.47***	−0.25	***0.68***	***−0.34***	***−0.32***	***−0.35***	***−0.35***
	CSF	−0.09	−0.19	***0.31***	***0.33***	0.19	−0.06	***0.47***	***0.45***	***0.48***	−0.06
	WBV	0.08	***0.42***	***−0.46***	***−0.51***	−0.20	***0.77***	***−0.65***	***−0.61***	***−0.67***	0.04

Note: Standardized regression coefficients estimated using generalized linear modeling to account for intracranial volume and age. Nominal p-values are reported in black typeface when tests are nominally significant (two-tailed a=0.05); *bold italicized* typeface shows which tests remained statistically significant upon adjusting for the false discovery rate. The colored bar figure shows the standardized regression coefficient (*β*) and shows its direction such that red shows negative associations, while blue shows positive relationships.

## Abbreviations

AD: axial diffusivity
AUC: area under the receiver-operating curve
COVID-19: coronavirus disease, 2019
FA: fractional anisotropy
FDR: false discovery rate
ISO: isotropic diffusion
MD: mean diffusivity
MRI: Magnetic Resonance Imaging
PASC: Post-Acute Sequelae of COVID-19
QA: Quantitative anisotropy
RD: radial diffusivity
T1-MPRAGE: T1-weighted magnetization-prepared rapid gradient echo
